# Delta Cell Hyperplasia in Adult Goto-Kakizaki (GK/MolTac) Diabetic Rats

**DOI:** 10.1155/2015/385395

**Published:** 2015-07-06

**Authors:** Lukáš Alán, Tomáš Olejár, Monika Cahová, Jaroslav Zelenka, Zuzana Berková, Magdalena Smětáková, František Saudek, Radoslav Matěj, Petr Ježek

**Affiliations:** ^1^Department No. 75, Institute of Physiology, Academy of Sciences, 14220 Prague, Czech Republic; ^2^Institute of Clinical & Experimental Medicine, 14021 Prague, Czech Republic; ^3^Teaching Thomayer Hospital and Third Medical School, Charles University, 14059 Prague, Czech Republic

## Abstract

Reduced beta cell mass in pancreatic islets (PI) of Goto-Kakizaki (GK) rats is frequently observed in this diabetic model, but knowledge on delta cells is scarce. Aiming to compare delta cell physiology/pathology of GK to Wistar rats, we found that delta cell number increased over time as did somatostatin mRNA and delta cells distribution in PI is different in GK rats. Subtle changes in 6-week-old GK rats were found. With maturation and aging of GK rats, disturbed cytoarchitecture occurred with irregular beta cells accompanied by delta cell hyperplasia and loss of pancreatic polypeptide (PPY) positivity. Unlike the constant glucose-stimulation index for insulin PI release in Wistar rats, this index declined with GK age, whereas for somatostatin it increased with age. A decrease of GK rat PPY serum levels was found. GK rat body weight decreased with increasing hyperglycemia. Somatostatin analog octreotide completely blocked insulin secretion, impaired proliferation at low autocrine insulin, and decreased PPY secretion and mitochondrial DNA in INS-1E cells. In conclusion, in GK rats PI, significant local delta cell hyperplasia and suspected paracrine effect of somatostatin diminish beta cell viability and contribute to the deterioration of beta cell mass. Altered PPY-secreting cells distribution amends another component of GK PI's pathophysiology.

## 1. Introduction

Type 2 diabetes mellitus (T2DM) is a complex metabolic disorder comprising both peripheral insulin resistance [1–4] and/or pancreatic beta cell dysfunction [2–5]. The impaired insulin responsiveness of peripheral tissues places increasing demands on insulin secretion. This may eventually lead to failure of beta cells. During the preclinical phase, pancreatic beta cells are still able to overcome the escalating insulin resistance, which may last for many years. Genetic predisposition and ongoing metabolic stress, lipid accumulation in the pancreas, glucagon overproduction, and beta cell structural damage and death as well as impairment of normal beta cell biogenesis lead to a gradual decline of the overall islet function. Also, an insufficiency for compensatory overproduction of beta cells in pancreatic islets (PI) due to beta cell dysfunction and/or loss of beta cell mass [3] may contribute.

Besides beta cells, PI are composed of alpha, delta, epsilon, and PP cells secreting glucagon, somatostatin (SST), ghrelin, and pancreatic polypeptide (PPY), respectively [4]. SST inhibits the release of insulin and glucagon in a paracrine manner [6]. All known beta cell secretagogues, such as glucose, arginine, gastrointestinal hormones, and tolbutamide, support the release of SST from delta cells [7]. PPY plasma levels increase with age as well as in both diabetes types [8]. But in type 2 diabetic patients, diet-induced weight loss and improvement of beta cell function are accompanied by a decrease in PPY plasma levels [9]. Recently, beta cell dedifferentiation into alpha cells has been suggested to participate in human type 2 diabetes etiology [10, 11]. A differentiation shift can arise when certain transcription factors diminish, like Nkx6.1, which controls a gene regulatory network required for establishing and maintaining beta cell identity [12, 13]. Also, the homeodomain transcription factor Hhex (hematopoietically expressed homeobox), required for delta cell differentiation, has been linked to type 2 diabetes and its deficiency released the paracrine inhibition of beta cell insulin secretion [14].

Due to multifactorial pathophysiological background of T2DM, different animal models have been developed to study predominantly only some of the underlying causes. A specific category of T2DM models represents those of polygenic origin that resemble in many aspects beta cells pathophysiology in human diabetes. Goto-Kakizaki (GK) rat strain represents one of the most frequently studied T2D models from this category [15]. The diabetic etiology in GK rats was suggested to include genetic contribution and gestational metabolic impairment inducing an epigenetic programming of the offspring transmitted over generations, causing reduced beta cell neogenesis and proliferation [16]. The prominent feature is the loss of beta cell differentiation related to chronic exposure to hyperglycaemia/hyperlipidaemia, islet inflammation, oxidative stress, fibrosis, and perturbed islet vasculature [15–18]. A striking morphologic feature of GK rat pancreatic islets lies in the existence of large islets with pronounced fibrosis due to separating strands of connective tissue and endocrine cells [19–21]. As a result, alpha and delta cells forming a mantle in nondiabetic rats are now spread within mostly the decreased beta cell mass [19–21]. The impaired glucose-stimulated insulin secretion (GSIS) is an invariant hallmark of GK rats independent of the type of colony [15–18, 22–24]. Since the elevated oxidative phosphorylation in mitochondria is the key component of the beta cell glucose sensor, findings of reduced amount [25, 26] of mitochondrial DNA are compatible with impaired beta cell mitochondrial function in GK rats [26]. Mitochondrial network was frequently fragmented in beta cells of GK rats, though mitochondrial volume was preserved [27]. Also disrupted microRNA regulation was found in GK rat beta cells [28].

Less attention has been paid to the role of other cell types, namely, delta cells. Previously, besides insulin secretion also the SST secretion as a response to glucose was found impaired in the perfused pancreas of GK rats [23]; hence we specifically focused on delta cells and SST PI content in fully developed diabetes. Surprisingly, we revealed a profound delta cell hyperplasia accompanied in elder animals by the increased glucose-stimulated SST release. Both of these factors may contribute to GK PI's pathophysiology.

## 2. Materials and Methods

### 2.1. Animals and Basic Tests

Goto-Kakizaki rats (GK/MolTac), derived from the inbred stock of Aarhus colony and bred randomly in a closed colony, and Wistar Han rats of the closest origin were purchased from Taconic Farms, Inc., Hudson, NY and were bred in accordance with the European Union Directive 2010/63/EU for animal experiments. All experiments were approved by the Institutional Animal Care and Use Committee of the Institute for Clinical and Experimental Medicine (Permit Number: 118361/2011-MZE-17214) and complied with the animal care protection law of the Czech Republic. Wistar rats were carefully selected according to their age to match with the corresponding Goto-Kakizaki rats ± two days. All surgery was performed under general anesthesia (dexmedetomidine 0.25–0.5 mg* per* kg body plus ketamine 50 mg* per* kg body weight), and all efforts were made to minimize suffering. Blood glucose was routinely estimated by an Accu-check Performa glucometer (Roche Diagnostics, Germany). For both intravenous glucose tolerance test (IVGTT) and intraperitoneal glucose tolerance test (IPGTT), the rats were starved overnight. IVGTT: rats were injected with 20% glucose into tail vein (1 g* per* kg body weight) and blood glucose was measured at the 10 min time intervals up to 60 min. IPGTT: rats were injected intraperitoneally a single dose of glucose (3 g* per* kg body weight). Blood was taken from tail vein at 15 or 30 min intervals up to 150 min.

### 2.2. Immunoassays

Determination of insulin, SST, and PPY was performed in serum from blood collected from the tail vein. The following kits were used according to manufacturer's protocols for evaluation of serum or cell culture media: (i) enzyme-linked immunosorbent assay (ELISA) Kit for Somatostatin E90592Ra (Life Technologies, Carlsbad, CA); (ii) Rat Pancreatic Polypeptide (PPY) Elisa kit E02P0134 (BlueGene Biotech, Shanghai, China); and (iii) Rat Insulin ELISA kits (Mercodia, Uppsala, Sweden).

### 2.3. Islets Isolation

PI isolation was performed always from a single rat according to a standard protocol [26, 27] in parallel from both groups, of age-matched GK and Wistar rats purchased specifically from the breeder (Taconic). GSIS was assayed in isolated PI during the following steps: (i) 15 min islet washing in basal medium with low glucose (3 mM), (ii) 60 min incubation in the basal medium, (iii) 60 min incubation after glucose addition to 22 mM final concentration, and (iv) return to the basal medium for 60 min. Aliquots were taken after each step and insulin content was tested by ELISA kit (Mercodia). Analogously, SST or PPY was assayed in the above described steps by the ELISA kits (Life Technologies or BlueGene Biotech, resp.). Samples for the SST assay had to be concentrated by acetone precipitation. For quantification of cell number in all assays, sample DNA content was estimated using Quant-iT PicoGreen ds DNA kit (Life Technologies) after islet lyses by SDS and proteinase K.

### 2.4. Tissue Samples

Whole 5% paraformaldehyde-fixed and paraffin-embedded samples of pancreas from Wistar and Goto-Kakizaki rats, typically of ages of 31, 52, and 79 weeks, were investigated using either monoclonal antibody mouse* anti*-insulin ab6995 (ABCAM, Cambridge, UK), diluted 1 : 100, or polyclonal antibodies such as rabbit* anti*-glucagon ab8055 (ABCAM), diluted 1 : 100, rabbit* anti*-somatostatin ab103790 (ABCAM), diluted 1 : 500, and rabbit* anti*-pancreatic polypeptide PA1-36141 (Pierce Biotechnology, Rockford, IL), diluted 1 : 1000. Secondary antibodies (Life Technologies), diluted 1 : 1000, were Alexa Fluor 568 Donkey* anti*-Mouse IgG, A10037 for red imaging, and Alexa Fluor 488 Donkey* anti*-rabbit IgG (H + L), A21206, for green imaging. Immunohistochemical samples were viewed by a motorized inverted fluorescence microscope Olympus IX-81 and Cell F software.

### 2.5. Peripheral Tissue Insulin Sensitivity/Resistance Test

The procedure was performed as described previously [30]. Briefly, rats were killed by decapitation and distal parts of epididymal adipose tissue (150 ± 25 mg) and diaphragm (160 ± 20 mg) were rapidly dissected. Note that aging GK rats exhibited up to four times less epididymal adipose tissue. The tissues were incubated in the absence or presence of 250 *μ*U/mL insulin for two hours in Krebs-Ringer bicarbonate buffer with 5 mmol/L glucose, 0.1 *μ*Ci (U-^14^C)-glucose* per* mL (UVVR, Řež, Czech Republic), and 2% bovine serum albumin, under 95% O_2_ and 5% CO_2_ at 37°C in sealed vials while shaking. Neutral lipids were extracted to chloroform/methanol (2 : 1, vol. to vol.) and the radioactivity was counted by scintillation counting. ^14^C-glucose accumulation into glycogen in diaphragm was determined after diaphragm digestion by boiling in 30% KOH and precipitation in 96% ethanol.

### 2.6. Cell Cultures

Rat insulinoma INS-1E cells were a kind gift from Professor Maechler, University of Geneva. 2·10^5^ INS-1E cells were maintained in 6-well test plates in 2 mL of total respective medium volume with daily replacement of 1 mL on days 1, 2, and 3 to maintain proper autocrine/paracrine relationship among insulin-secreting cells. Each group consisted of 6 samples, and in treatment group octreotide (Sandostatin, Novartis, Basel, Switzerland) in working concentration of 1 *μ*M was added every day on days 0, 1, 2 and 3. On day 4, the media was collected and processed by ELISA immunoassay.

### 2.7. RT PCR

Primers were designed using Lasergene Genomic Suite software (DNASTAR, Madison, WI) for rat SST: 5′-CCT GGC TTT GGG CGG TGT CA-3′ (forward); 5′-CTC AGG CTC CAG GGC ATC GTT CT-3′ (reverse); and for rat beta actin: 5′-CCA CAC CCG CCA CCA GTT CG-3′ (forward); 5′-GGC CCG GGG AGC ATC GTC-3′ (reverse). The PCR reaction was performed in LightCycler 480 (Roche) utilizing Maxima SYBR Green qPCR Master Mix (Pierce Biotechnology, Meridian Road Rockford, IL). The absolute mRNA amounts were calculated from crossing points of each run. Copy number of mitochondrial DNA (mtDNA)* per* cell has been assayed as described previously [26].

### 2.8. Statistical Analysis

Data are presented as mean ± s.d., while ANOVA with Tukey test on the prevalidated data through a normality test or Student's *t*-tests (two samples) were used for statistical analyses.

## 3. Results 

### 3.1. Delta Cell Hyperplasia in Adult Goto-Kakizaki Diabetic Rats

**Figure 1:**
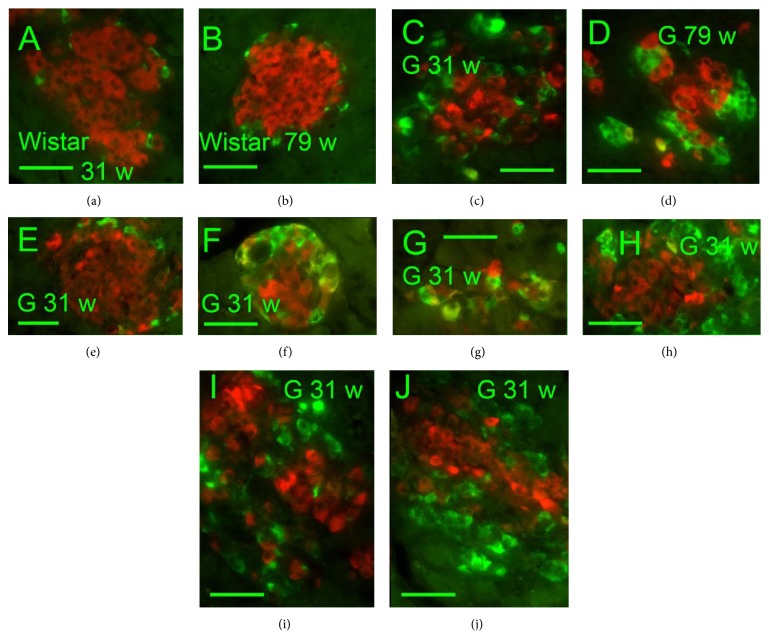
Insulin and somatostatin immunohistochemical pattern of pancreatic islets.* Insulin: red*,* SST*:* green*, and “w”: weeks. Overlay images of pancreatic islets of 31- and 79-week-old Wistar (a, b) and Goto-Kakizaki (c–j) rats “G.” Individual or a few SST-positive delta cells were observed in Wistar controls of both ages (31 and 79 weeks) (a, b). In GK rats, increased number of strongly positive delta cells containing SST was recorded predominantly in the PI periphery as well as strongly irregularly in both ages of 31 (c, e–j) and 79 weeks (d). Original magnification 400x; scale bars 50 *μ*m.

**Figure 2:**
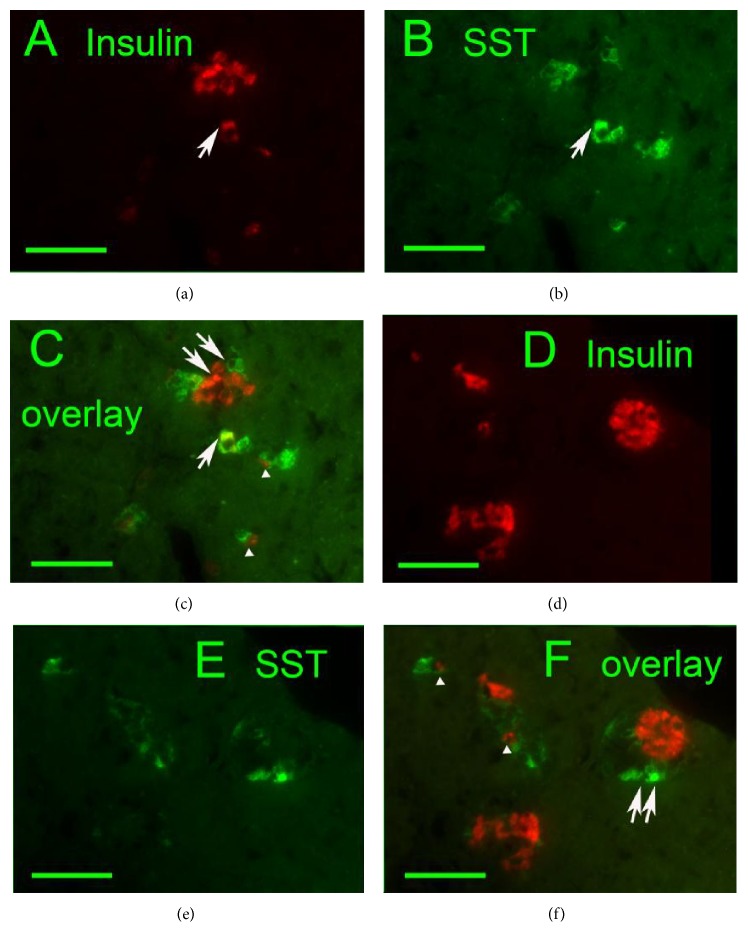
Delta cell proliferation in exocrine glandular tissue ((a), (d)* insulin*:* red*, (b), (e)* SST*:* green*, and (c), (f) overlays) of 79-week-old Goto-Kakizaki rats. Individual exocrine cells present simultaneous content of insulin and SST (arrows). Coupling of individual SST- and insulin-positive endocrine cells was frequently noticed (arrowheads). Image of small proliferating endocrine masses indicates also beta and delta cells polarization (double arrows). Original magnification 400x; scale bars 50 *μ*m.

**Figure 3:**
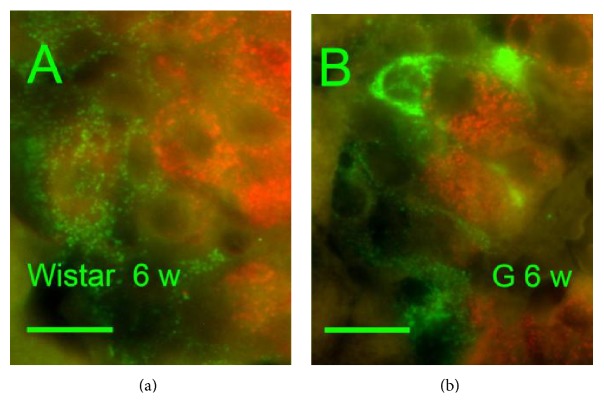
Insulin and somatostatin immunohistochemical pattern in six-week-old rats.* Insulin*:* red*,* SST: green*, and “w”: weeks. Overlay images of pancreatic islets of six-week-old Wistar (a) and Goto-Kakizaki (b) rats “G.” Only a fine granular SST positivity was observed in Wistar controls, whereas, in GK rats, individual or small groups of cells with irregular rough and dense SST positivity could be recorded. Original magnification 1000×; scale bars 12.5 *μ*m.

**Figure 4:**
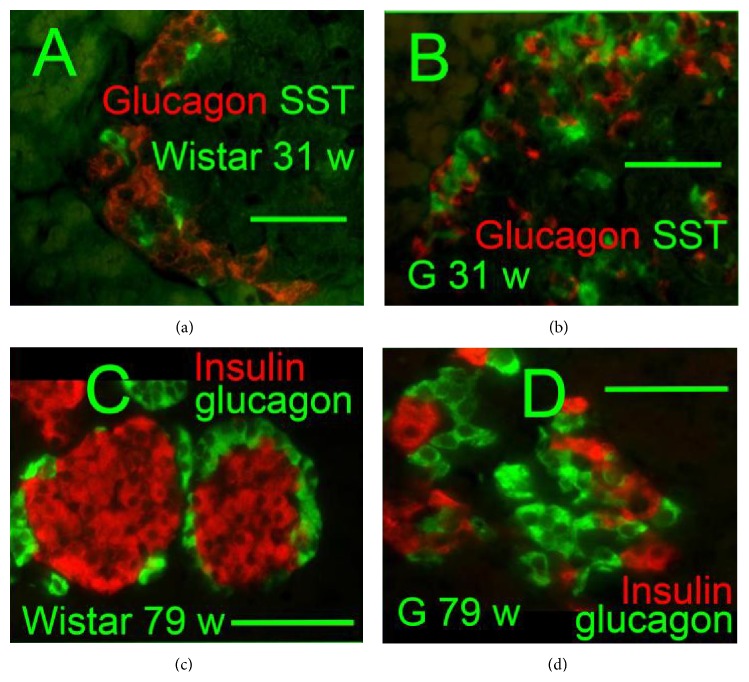
Glucagon, somatostatin, and insulin immunohistochemical pattern of pancreatic islets (a), (b)* glucagon*:* red* and* SST*:* green*; (c), (d)* insulin*:* red*,* glucagon*:* green*, and “w”: weeks. Overlay images of pancreatic islets of 31-week-old Wistar (a) and Goto-Kakizaki (b) rats “G” and 79-week-old Wistar (c) and Goto-Kakizaki (d) rats “G.” Original magnification is 400x in (a), (b) and 600x in (c), (d); scale bars 20 *μ*m.

**Figure 5:**
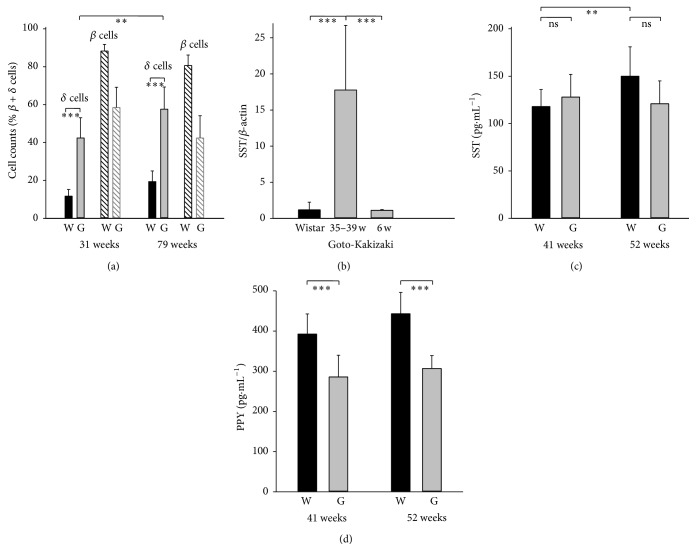
Delta cell* versus* beta cell counts and somatostatin pancreatic and serum levels plus PPY serum levels in Wistar and GK rats. (a) Cell counts from immunohistochemical PI sections of Wistar “W” (*n* = 6 samples) and GK rats “G” (*n* = 9 samples) as % fraction of beta plus delta cells for cells as specified at the age of 31 and 79 weeks (^*∗∗*^ *p* < 0.01; ^*∗∗∗*^ *p* < 0.001). (b) SST mRNA quantification as the relative SST/beta actin ratio in PI of Wistar (black bar) and GK rats (gray bars) indicates ~14-fold increase of SST mRNA. Five estimates (*n* = 5) at weeks 6 and 35, 37, or 39 (^*∗∗∗*^ *p* < 0.001). (c) SST and (d) PPY in serum collected from the tail vein of Wistar “W” (black bars) and Goto-Kakizaki “G” (gray bars) rats aged 41 and 52 weeks (^*∗∗∗*^ *p* < 0.001 (*n* = 11); for Wistar SST otherwise *n* = 6).

Insulin-, glucagon-, and somatostatin- (SST-) positive cells were screened immunohistochemically in isolated PI (Figures 1–4). A typical morphology pattern for insulin-positive cells was recorded in PI of Wistar controls (Figures 1(a), 1(b), 2(a), and 4(c)), as well as for glucagon-positive cells (Figures 4(a) and 4(c)). In GK rats, a disturbed cytoarchitecture was observed predominantly in elder animals with irregular beta cells of nonhomogenous and irregular insulin positivity (Figures 1(c)–1(j)) or irregular alpha cells and glucagon positivity (Figures 4(b) and 4(d)). Individual or a few SST-positive delta cells were observed in 31- and 79-week-old Wistar controls (Figures 1(a), 1(b), and 5(a)). In 31-, 52-, and 79-week-old GK rats, the increased number of strongly SST-positive cells was observed (Figure 5(a)) in an irregular pattern and in the periphery of PI (Figures 1(c) and 1(d)). When quantified (Figure 5(a)), a 10–20% of the overall counts of beta plus delta cells in PI sections found in young and old Wistar rats were significantly increased in GK rats up to 60%. For 31-week-old GK rats the histological pattern ranged from a very rarely observed normal pattern with only a few SST-positive cells in the islet periphery,* via* abundant SST-positive wide layers in the islet periphery, to more and more irregular pattern with predominant cell SST positivity (Figures 1(e)–1(j), 4(a), and 4(b)). In 52- and 79-week-old GK rats, no intact histological features such as those found in Wistar control islets were observed, but only SST-positive layers in the islet periphery with predominant irregular SST locations.

The SST and insulin coexpression was also recorded. Contrary to Wistar rats, 31-, 52-, and 79-week-old GK rats were contained in their PI small proliferating nests and individual cell groups, exhibiting both SST- and insulin-positivity. The clearly visible SST and insulin coexpression was observed mostly in smaller endocrine masses dispersed in the exocrine glandular tissue, predominantly in elder animals (Figure 2). No coexpression of SST and glucagon was observed (Figures 4(a) and 4(b)).

Investigating young 6-week-old rats, we found that the number of SST-positive delta cells was not higher in GK rats* versus* Wistar controls (15% ± 3% of summary cell counts in GK rats* versus* 18% ± 5% in Wistar rats). Six-week-old Wistar rats exhibited PI with only a fine granular SST positivity (Figure 3(a)), whereas six-week-old GK rat PI contained individual or small groups of cells with irregular rough and dense SST positivity (Figure 3(b)). The content of insulin-positive cells was not altered in young GK rats in comparison to young Wistar controls (85% ± 3% of all cells of a section in GK rats* versus* 80% ± 5% of all cells in Wistar rats).

### 3.2. Factors Accompanying Delta Cell Hyperplasia

The expression of SST-specific mRNA paralleled the histological findings. The RT PCR assessment (relatively to beta actin) in isolated PI of 35–39-week-old rats has shown a ~14-fold increase of SST mRNA (Figure 5(b)) in GK rats as compared to Wistar controls. Despite the fact that it may include not only delta cell enumeration but also the increased SST gene expression, this result complies with the histological data. Young, six-week-old GK rats exhibited unchanged, that is, low levels, of SST mRNA (Figure 5(b)). ELISA determination of SST in serum collected from the tail vein of 41- and 52-week-old rats showed no statistically significant difference (Figure 5(c)). This result is expected, since peptide hormone SST has a relatively short half-life and is degraded rapidly in plasma and is also synthesized plus being secreted by neuroendocrine cells in the central nervous system and the gastrointestinal system, while the latter is the major contributor to circulating SST.

**Figure 6:**
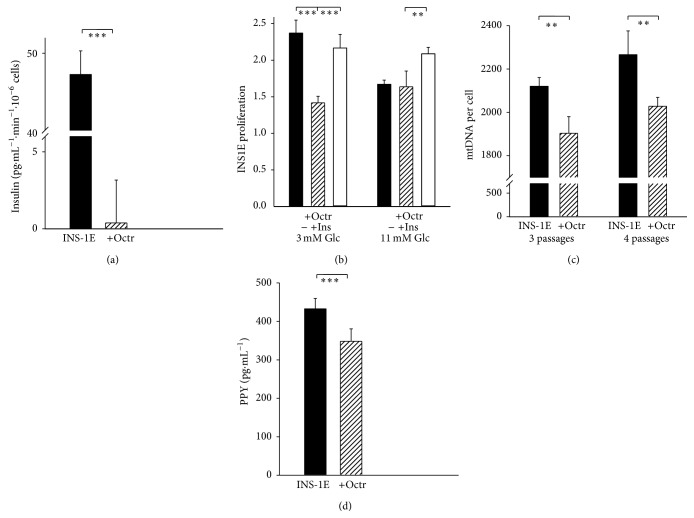
Octreotide effect on insulin release, proliferation, mtDNA, and PPY secretion of INS-1E cells. (a) Glucose-stimulated insulin release from INS-1E cells, evaluated as the difference between time courses assayed with 25 mM glucose and without glucose up to 30 min, in the absence (black bar) and after a 60 min pretreatment with 50 nM octreotide (dashed bar); ^*∗∗∗*^ *p* < 0.001 (*n* = 3). (b) Proliferation (relative number of cells related to zero time)* of INS-1E cells* for 3 various cultivations at 3 mM glucose “3 mM Glc”, that is, at suppressed autocrine insulin, or at standard 11 mM glucose, in the presence of 10 nM octreotide (dashed bars) or octreotide plus supplemented 0.2 *μ*M human insulin “+Ins” (white bars, ^*∗∗*^ *p* < 0.01; ^*∗∗∗*^ *p* < 0.001); (c) mitochondrial DNA copy number in INS-1E cells, after 3 or 4 passages “pass.” of cultivation in the absence (black bars) or presence of 1 *μ*M octreotide (dashed bars, ^*∗∗*^ *p* < 0.05; *n* = 6). (d) PPY in medium of control (black bar) and octreotide-treated (1 *μ*M, +Octr, dashed bar) INS-1E cells, after 3 passages of cultivation (^*∗∗*^ *p* < 0.05; *n* = 6).

An SST-receptor agonist octreotide (50 nM, 60 min pretreatment) blocked 99% of the glucose-stimulated insulin release in model beta cells, INS-1E cells, assayed for 30 minutes after glucose addition to 25 mM (Figure 6(a)). Octreotide also decreased proliferation of cultured INS-1E cells, however, only in the absence of autocrine insulin, that is, in cultivations with 3 mM glucose (Figure 6(b)), where autocrine insulin in cell culture is not sufficient. In turn INS-1E cell proliferation was not affected by octreotide at routine cultivation with 11 mM glucose promoting autocrine insulin (Figure 6(b)). Also, 1 *μ*M octreotide after 3-4 cultivation passages decreased mitochondrial DNA copy number in INS-1E cells by more than 10% (Figure 6(c)).

### 3.3. Pancreatic Polypeptide in Adult Goto-Kakizaki Diabetic Rats

**Figure 7:**
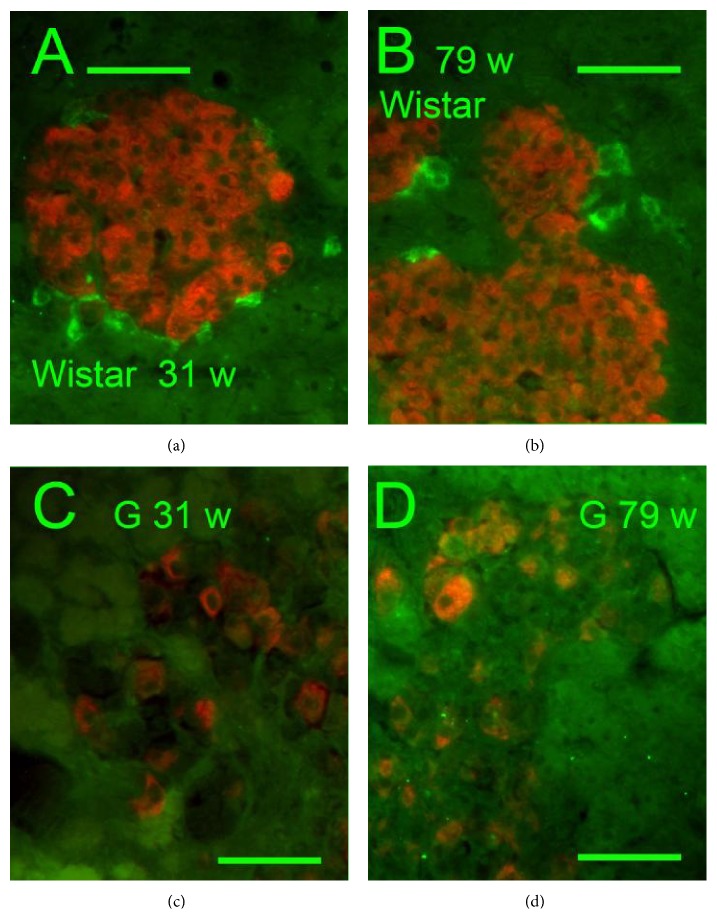
Insulin and PPY immunohistochemical pattern of islets.* Insulin*:* red*,* PPY*:* green*,* and *“*w*”:* weeks*. Overlay images of PI of Wistar (a, b) and Goto-Kakizaki rats (c, d). Common pattern of PPY positive cells was observed in Wistar controls of both ages of 31 (a) and 79 weeks (b). In GK, no PPY positive cells were seen in both ages of 31 (c) and 79 weeks (d). Original magnification 600x; scale bars 50 *μ*m.

Individual or a few PPY-positive cells were observed in 31- and 79-week-old Wistar rats (Figures 7(a) and 7(b)). In age-matched GK rats, no PPY-positive cells were observed (Figures 7(c) and 7(d)). Nevertheless, no difference of PPY on mRNA levels was observed between GK and Wistar rats (data is not shown). In turn, significantly lower values were recorded for PPY serum levels in 41- and 52-week-old GK rats as compared to the age-matched Wistar controls (Figure 5(b)). Also significantly lower PPY values were recorded even for INS-1E cells treated with 1 *μ*M octreotide for 4 days (Figure 6(d)).

### 3.4. Circulating Insulin Levels and Glycemia in GK Rats

**Figure 8:**
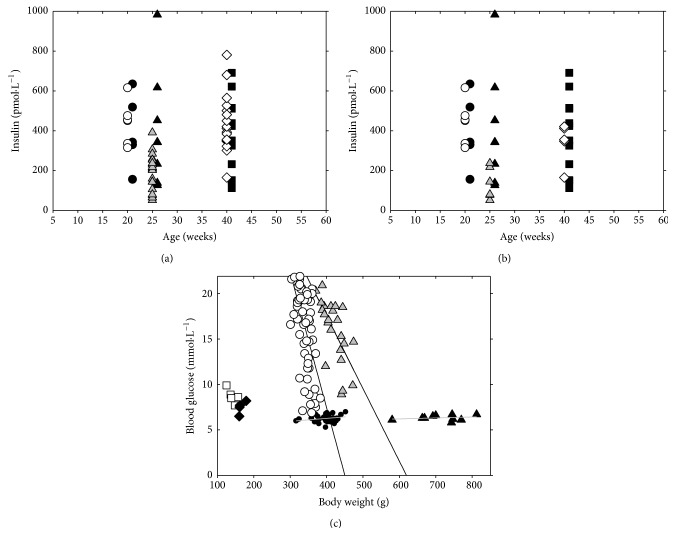
Postprandial circulating insulin levels and relationships between fasting blood glucose and body weight during maturation and aging of Wistar and Goto-Kakizaki rats.* Wistar rats*:* black symbols*;* GK rats*:* gray and white symbols*. Postprandial insulinemia in blood of (a) all studied GK rats* versus* Wistar rats; (b) hyperglycemic GK rats were selected* versus* all studied Wistar rats at age as indicated; and (c) relationship of blood glucose in a fasting state on body weight at the age of six weeks (white squares; black diamonds), 15 weeks (circles), and 37 weeks (triangles).

We have carefully investigated postprandial insulinemia (Figures 8(a) and 8(b)) and fasting hyperglycemia (Figure 8(c)) in GK rats of various ages. In 20- and 40-week-old rats of our GK/MolTac colony, the circulating insulin levels in fed animals were spread in a wide range as those in age-matched Wistar rats (Figure 8(a)). However, when selecting only values for hyperglycemic GK rats at fasting state, lower and statistically distinct circulating insulin levels were accounted (Figure 8(b), Table 1). This stems from a great heterogeneity among the studied GK rat group, consisting of hyperglycemic, mild hyperglycemic, and nearly normoglycemic animals (Figure 8(c), Table 1), dependent on their weight. The average glucose levels in Wistar rats were normoglycemic and constant between weeks 15 and 37. Young, six-week-old GK rats had slightly but nonsignificantly elevated glycemia, which elevated on average to 15.9 ± 3.4 mM at week 37 (Figure 8(c), Table 1). The average weight increased in Wistar rats from 496 ± 21 g to 712 ± 66 g and in GK rats from 342 ± 19 g to 420 ± 28 g. Consequently, no relationship between weight and glucose level was observed in Wistar rats, whereas a decrease of glucose levels with increased weight was characteristic for GK rats (Figure 8(c)). All these data not only indicate that the diminished beta cell amount in GK rats is sufficient to supply insulin, even if at diminished levels in some individual animals, but also reflect that the completely developed insulin resistance in GK rats prolongs the half-time of blood circulating insulin.

### 3.5. Insulin Resistance in GK Rats

**Figure 9:**
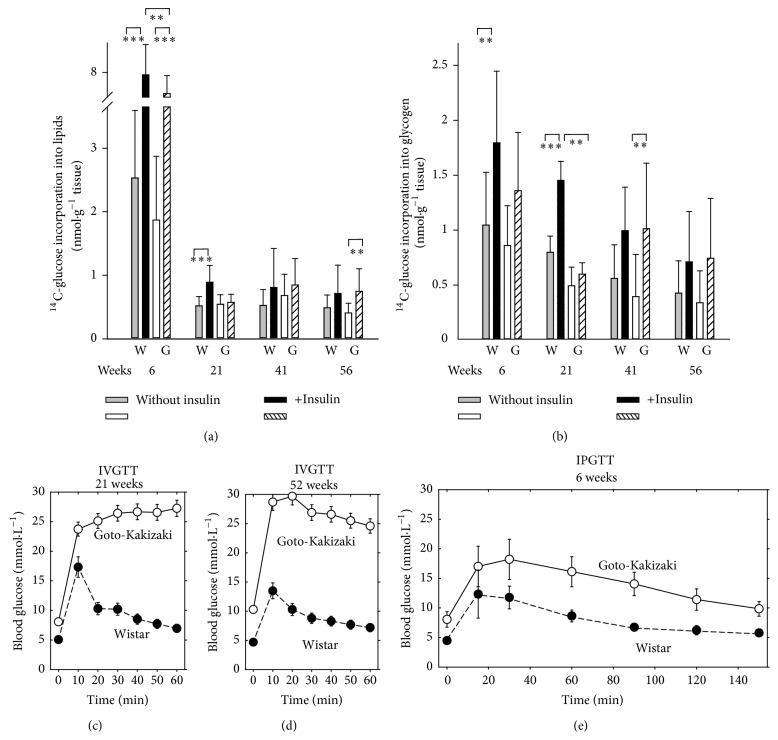
*In vitro* insulin sensitivity (a, b) and* in vivo* insulin resistance (c, e) of Goto-Kakizaki rats. (a) and (b) ^14^C-glucose incorporation into lipids (a) of epididymal adipose tissue and into glycogen of diaphragm (b);* in vitro* data obtained in the absence (*gray* or* white bars*) or presence of an insulin stimulus (*black* or* dashed bars*) in four selected age groups at indicated week of age are plotted for Wistar “W” and Goto-Kakizaki rats “G.”  ^*∗∗*^ANOVA yielded *p* < 0.05 from the following ensembles: *n* = 18–20 in (a) for 6 and 41 weeks, *n* = 9–12 in (a) for 6 and 41 weeks, and *n* = 8–10 in (b). (c)–(e) Intravenous glucose tolerance test “IVGTT” or (f) intraperitoneal glucose tolerance test “IPGTT” during aging of Wistar and Goto-Kakizaki rats. Blood glucose levels are indicated within the given time course after addition of glucose (see Section 2.1 for details) for a group of six rats of the given age as indicated.

Hence, next we tested the peripheral insulin resistance as the insulin-stimulated glucose incorporation into lipids (adipose tissue) (Figure 9(a)) and glycogen (skeletal muscle) (Figure 9(b)). 21-week-old GK rats exhibited complete insulin resistance in both adipose tissue and skeletal muscle while Wistar rats retained normal insulin sensitivity. In older animals of both strains the response to insulin in both tissues was low and no significant differences were found in incorporations prior to and after insulin dosage. The IVGTT test more clearly distinguished within the complete population and age groups of GK and Wistar rats and indicated that since week 21, GK rats were nearly completely glucose intolerant while in Wistar rats glucose tolerance remained stable (Figures 9(c)–9(e)). All Wistar rats of all tested age groups exhibited the glucose clearance peak at ~10 min (20 min for the intraperitoneal test at week 6), whereas GK rats exhibited a very low-glucose clearance, keeping high glucose permanent up to 60 min (Figures 9(c)–9(e)). The six-week-old GK rats exhibited prolonged decline of glucose, apparently shifting its clearance peak to a longer time, distinguishing them from older GK rats (Figure 9(e)).

### 3.6. Decreasing Glucose-Stimulated Insulin Secretion versus Enhanced Glucose-Stimulated SST Secretion in GK Rats during Aging

**Figure 10:**
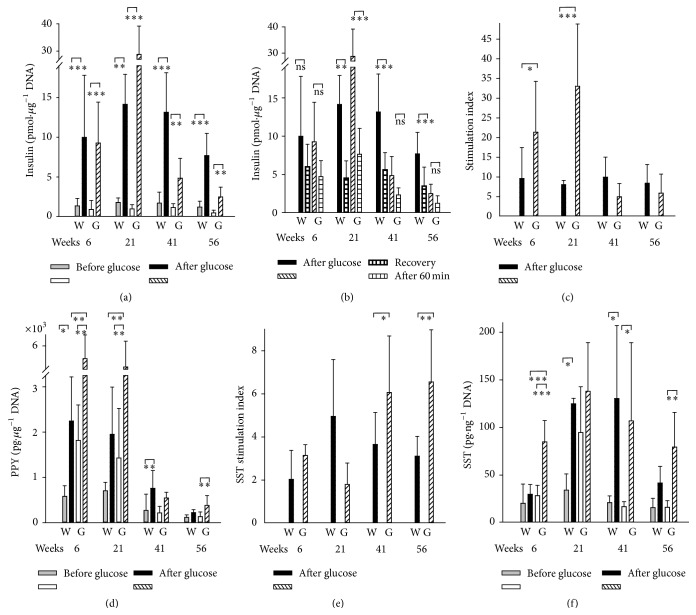
Glucose-stimulated insulin (a–c), PPY (d), and SST secretion (e, f) from isolated PI of Wistar and Goto-Kakizaki rats during aging. GSIS or glucose-stimulated secretion of PPY or SST was assayed for isolated PI of Wistar “W” and Goto-Kakizaki rats “G” of age in weeks indicated by numbers. Panels (a), (d), and (f) show* insulin*,* PPY*,* or SST*, respectively, in pmol* per* *μ*g DNA, pg* per* *μ*g DNA, or pg* per* ng DNA, released* before* (gray or white bars) and* after glucose addition* (black or dashed bars), while panel (b) repeats displays of insulin released after glucose addition (black or dashed bars) and compares them with magnitudes of a “*recovery*” established 60 min after the return into the low-glucose basal medium (horizontally dashed bars), and panels (c) and (e) show the calculated* insulin* (c)* or SST* (e)* glucose-stimulation index* as ratio of insulin or SST, respectively, released after* versus* that before glucose addition. ANOVA yielded ^*∗∗∗*^ *p* < 0.001, ^*∗∗*^ *p* < 0.05, or ^*∗*^ *p* < 0.1; from the following ensembles: *n* = 10–14 in (a–c) (5–9 for 56 weeks), *n* = 5–8 in (d), and *n* = 5 in (e, f).

Despite the reduced beta cell content in GK rat PI and despite a possible recovery during* in vitro* cultivation of isolated PI, we did not find any fatal reduction of* in vitro* glucose-stimulated insulin secretion in isolated PI (GSIS_*in* *vitro*_) (Figures 10(a)–10(c)). The insulin stimulation index was even significantly higher for 6- and 21-week-old GK rats, while it drastically declined at high age, but insignificantly below the levels found in age-matched Wistar rats (Figure 10(c)).

Also, glucose-stimulated PPY secretion of isolated islets was higher for 6- and 21-week-old GK rats, when compared to the age-matched Wistar rats (Figure 10(d)). The subsequent decline of glucose-stimulated PPY secretion in higher age was equal at 41-week-old GK rats and was even lower at 56-week-old GK rats* versus* the age-matched Wistar rats (Figure 10(d)) [8].

In accordance with the observed delta cell hyperplasia, the SST stimulation index for the glucose-stimulated SST secretion in isolated PI was increasing with age of GK rats and was significantly higher when compared to the age-matched Wistar rats (41- and 56-week-old) (Figure 10(e)). Glucose-stimulated SST secretion from GK PI* in vitro* did not decline with age as intensively as GSIS (cf. Figures 10(f) *versus* 10(a)); and for 56-week-old GK rats, glucose-stimulated SST secretion was higher than for 56-week-old Wistar rats. Despite aging contribution to the decreased secretion of all, insulin, PPY, and SST in isolated PI in Wistar rats, we can conclude that in GK rats there are a much lower decline of SST secretion and high decline of insulin secretion, reflecting the status of beta cell reduction at the simultaneous delta cell hyperplasia.

## 4. Discussion

### 4.1. Characteristics of Goto-Kakizaki Rat Model

The Goto-Kakizaki rat represents one of the best characterized animal models of spontaneous type 2 diabetes [15–28]. The line was established by repeated breeding of Wistar rats with glucose tolerance at the upper limit of normal distribution. Its main characteristics include fasting hyperglycemia and impaired insulin secretion in response to glucose together with a variable rate of insulin peripheral resistance in the absence of obesity [15–18, 22–24]. Typically, pancreatic islets lose their characteristic architecture with progressive beta cell loss and fibrosis [16]. Though altered islet localization of alpha and delta cells together with slightly higher islet somatostatin content has been demonstrated [23], extensive delta cell hyperplasia has not been reported in previous studies and its potential significance for diabetes development has not been discussed so far.

### 4.2. Delta Cell Hyperplasia as Novel Revealed Feature

In the present study we bring new evidence that the local SST overproduction by propagated delta cells with potential contribution of the loss of PPY producing cells is also involved in beta cells dysfunction and gradual attrition in this model. We have observed hyperplasia of somatostatin-positive delta cells, accompanied by the increased SST glucose-stimulation index of PI* in vitro*, together with the increased amounts of SST mRNA in PI of aging Goto-Kakizaki rats. Delta cell hyperplasia developed after six weeks of age (Figures 5(a), 5(b), and 10(f)). Nevertheless, the peripheral insulin resistance was already partly developed in six-week-old GK rats (Figure 9(e)) and existed with ~100% occurrence in GK rats older than six weeks.

Our new findings may hypothetically reflect two lines of contribution of delta cell hyperplasia to the developing diabetic etiology: (i) SST effects deteriorating beta cell viability at the simultaneously diminished autocrine insulin maintenance of beta cells [31] and (ii) acceleration of the already altered (impaired) biogenesis of beta cells due to delta cell hyperplasia. However, the most realistic view could be that these lines act in a synergy. In addition, mutual pathology-accelerating interactions with the early developed peripheral insulin resistance should promote diabetic etiology.

Impairment of the paracrine interrelationships must originate from the observed increasing SST glucose-stimulation index (as found in PI* in vitro*). Its higher values are expected as the direct consequence of the revealed delta cell hyperplasia. The additional pathology originates from the well-known disordering of the alpha and delta cell mantle in PI of GK rats [19–21]. Within the broken islet, scattered beta cells are no longer functionally synchronized, as within the ordered PI core of intact islets forming a membrane potential* syncytium*. Expected long-lasting local SST overproduction by enumerating delta cells and the concomitant enhanced paracrine SST effect on the diminishing number of beta cells with deteriorating autocrine insulin function [31], which is otherwise supporting beta cell viability, must affect normal beta cell maintenance. There are several possible targets of SST action. Grozinsky-Glasberg et al. [32] have demonstrated that octreotide inhibited Akt/mTOR/p70S6K pathway in INS-1 cells and independently downregulated cell proliferation. As we found for INS-1E cells, SST lowered mitochondrial DNA and affected viability of beta cells only in the absence of beneficial autocrine insulin maintenance. Albeit insulin stimulation index declines with age similarly in GK and Wistar rats, only for GK SST may thus act as a factor additionally decreasing the beta cell mass. In conclusion, our data suggest that paracrine SST deterioration of beta cell viability at declining autocrine insulin function [31] may contribute to GK rat diabetic etiology* in vivo*.

The previously reported reduced glucose-induced SST release [23] had to originate due to the aging effect, confirmed now in this work. The declining glucose-induced SST release [23] may also originate from the disruption of cell-to-cell contacts [33]. In contrast, we have observed higher SST release in 56-week-old GK rats* versus* Wistar controls. A reduced insulin secretion, at least due to the diminishing number of beta cells, could not influence SST secretion [34].

### 4.3. PPY-Related Pathology of GK Rats

The lack of PPY-positive cells in aging GK rat PI [35] represents an additional pathogenic component.* In vitro* PPY secretion in isolated islets upon glucose stimulus is equally reduced during aging of Wistar and GK rats. The decreased PPY in serum of GK rats may suggest a possible PP cell inhibition by overproduced SST [36, 37], similarly to the observed decline in PPY secretion in INS-1E cells after the octreotide treatment. In turn, PPY production is enhanced at insufficient SST [38]. Overall, age-related decline of glucose-stimulated insulin and PPY secretion exhibits simply a higher extent than a decline in SST secretion.

### 4.4. Possible Origin of Delta Cell Hyperplasia

The evidence for the altered biogenesis of beta cells, delta cells, and PP cells in GK rats was suggested by immunohistochemistry, showing the simultaneous presence of SST- and insulin-positive cells in small endocrine masses (not seen in Wistar controls). This may indicate a certain differentiation shift from beta to delta cells, supported also by the clearly visible SST and insulin coexpression in individual endocrine cells dispersed within the exocrine glandular tissue. Further support is given by the existence (yet scarce) of coupling of individual SST- and insulin-positive cells as well as by certain levels of beta and delta cells polarization in small endocrine masses (Figure 2). This suggests a speculation considering a possible splitting of a common beta/delta progenitor into two separated cells, where the delta component continues in an uncontrolled proliferation leading to the delta cells hyperplasia as observed in the fully developed disease in aged GK rats.

**Figure 11:**
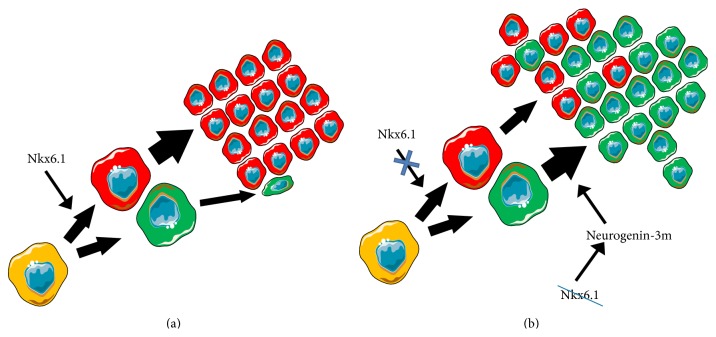
Hypothetical origin of delta cell hyperplasia. Schema was composed on the basis of [12, 13, 29] (see Section 4). (a) In nondiabetic animals, such as Wistar rats, SST-containing delta cells terminate proliferation after splitting from a common beta/delta cell progenitor while insulin-positive beta cells proliferate. (b) On the contrary, in diabetic Goto-Kakizaki rats, delta cells proliferation is not terminated, thus forming masses of SST positivity by the beta cell core mass. Origin of clonal proliferation of cells after splitting from the beta/delta cell progenitor can result from the known decreasing beta cell mass in Goto-Kakizaki PI, logically accompanied by the decrease of the beta cell-specifying factor Nkx6.1. A reduction of Nkx6.1 has been reported to increase neurogenin-3m, which in the absence of other factors promotes delta cells [29]. Common beta/delta progenitor cell: yellow, beta cells: red, and delta cells: green. The scheme was composed using Servier Powerpoint Image Bank: http://www.servier.com/.

Since the glucose-stimulation index for SST* in vitro* increased with age of GK rats, being nearly constant in nondiabetic controls, whereas the glucose-stimulation index for insulin rapidly declined with age in both diabetic GK rats and Wistar controls, this clearly reflects new relationships set by the delta cell hyperplasia. The common progenitors are well known for pancreatic cell types as well as transdifferentiation of exocrine acinary cells leading to the islet regeneration [4, 39–42]. One may speculate (Figure 11) that such dissociation between beta and delta cells may originate from the lack of factors essential for maintaining the functional state of pancreatic beta cells, such as transcription factor Nkx6.1 [12, 13]. It has been reported that when Nkx6.1 is deleted in beta cells, beta cells convert into delta cells, but not into alpha or pancreatic polypeptide- (PP-) producing cells [12]. Moreover, a decreased Nkx6.1 expression induces neurogenin-3 expression [13]. Neurogenin-3 possesses a critical role in establishing a generic endocrine state in acinar cells, which stimulates transdifferentiation into delta cells [29].

Hypertrophy and hyperplasia of delta cells were previously reported in juvenile type of diabetes mellitus in humans and streptozotocin-induced diabetes in rats [43] and in diabetic mice [44, 45]. A 56% elevation of SST in GK pancreata compared to Wistar controls has been reported [23], complying with our finding of the increase of the SST glucose-timulation index. Taken together with the reciprocal dependence of hyperglycemia on body weight of GK rats, observations of delta cell hyperplasia with SST overproduction raise doubts whether GK rats represent a typical canonical type 2 diabetes model, considered as originating from a beta cell failure due to apoptosis [10]. In turn, a significant diabetic etiology component is manifested in GK rats, which much more resembles secondary diabetes mellitus observed in somatostatinoma or states after treatment with somatostatin analogs [46, 47]. However, similarities can be found even to human diabetic islet and pancreas morphology changes [48]. Interestingly, delta cell fraction and area remained unchanged in cadaveric pancreatic sections from patients of type 2 diabetes [48]. They otherwise exhibited a preferential loss of large islets, with decreasing beta cell fraction and a reciprocal elevation in alpha cell fraction [48]. Nevertheless, the total alpha cell area was diminished [48].

## 5. Conclusions

In conclusion, GK rats develop a type 2 diabetes with a pronounced either primary or secondary hyperplasia of somatostatin-positive delta cells. This suggests a possible role of somatostatin component in pathogenesis of this animal model. A reduction of immunohistochemical PPY positivity was also observed. The enumerating delta cells represent a hallmark of the newly revealed GK rat phenotype. This finding resembles the recently suggested beta cell dedifferentiation into alpha cells for human type 2 diabetes [10].

## Figures and Tables

**Table 1:** Postprandial insulinemia in hyperglycemic rats and fasting glycemia in all rats within studied groups.

	Goto-Kakizaki rats			Wistar rats		
	20 weeks	25 weeks	42 weeks	20 weeks	25 weeks	42 weeks
Insulinemia (pmol·L^−1^) Average ± SD (median)	**442** ± 99 (453)	**134** ± 71 (112) *p < 0.05 *	**340** ± 92 (355)	**386** ± 153 (337)	**404** ± 266 (343)	**401** ± 171 (424)

	6 weeks	15 weeks	37 weeks	6 weeks	15 weeks	37 weeks

Glycemia (mmol·L^−1^) Average ± SD (median) [*n*]	**8.7** ± 0.7 (8.6) [*n* = 6]	**16.2** ± 4.6 (17.2) [*n* = 90] *p < 0.001 *	**15.9** ± 3.4 (17.1) [*n* = 23] *p < 0.001 *	**7.5** ± 0.6 (7.6) [*n* = 6]	**6.3** ± 0.4 (6.3) [*n* = 10]	**6.3** ± 0.3 (6.3) [*n* = 30]

Statistical significance between age-matched groups of Goto-Kakizaki *v*ersus Wistar rats is noted. For insulinemia estimations *n* = 6.
